# Cost Comparison of Percutaneous Nephrolithotomy With and Without Intraoperative Cone-beam Computed Tomography: 18-month Postoperative Analysis

**DOI:** 10.1016/j.euros.2025.12.002

**Published:** 2025-12-18

**Authors:** Rosanne van Ee, Chris A. Suijker, Antoinette D.I. van Asselt, Inge M. van Oort, Riemer A. Kingma, Stijn Roemeling

**Affiliations:** a Department of Urology, University Medical Center Groningen, University of Groningen, Groningen, The Netherlands; b Department of Health Sciences, University Medical Center Groningen, University of Groningen, Groningen, The Netherlands; c Department of Epidemiology, University Medical Center Groningen, University of Groningen, Groningen, The Netherlands

**Keywords:** Percutaneous nephrolithotomy, Intraoperative cone-beam computed tomography, Cost comparison, Stone-related events, Randomized controlled trial, Urolithiasis, Residual fragments

## Abstract

**Background and objective:**

Residual fragments (RFs) after percutaneous nephrolithotomy (PCNL) increase the risk of stone-related events (SREs) such as reinterventions and emergency department (ED) visits. Intraoperative cone-beam computed tomography (CBCT) facilitates detection and removal of RFs to improve stone-free rates and potentially reduce SREs. To determine whether the initial increase in surgical costs for CBCT in a hybrid operating room (OR) is offset by a reduction in overall expenses by minimizing SREs, we compared the total in-hospital health care costs of standard PCNL versus CBCT-PCNL over an 18-mo period.

**Methods:**

Data from a previous randomized controlled trial including 80 patients undergoing CBCT-PCNL and 80 undergoing conventional PCNL were analyzed. Procedural costs were calculated by multiplying operative duration by the Dutch reference price per minute, and adding disposable costs. Follow-up costs included costs for complications, SREs (reinterventions, ED visits, drainage, admissions), imaging, and consultations during 18 mo.

**Key findings and limitations:**

Assuming utilization rates of 42% for a hybrid OR and 92% for a conventional OR and following reference prices, we calculated mean total costs per patient of €8725 for the CBCT group and €8564 for the control group, with a difference of €167. The 40.2% higher procedural costs for hybrid-OR PCNL were nearly offset by 38.3% lower follow-up, complication, and SRE costs. Limitations include the single-center design, incomplete cost standardization, and the exclusion of non-hospital costs such as productivity loss.

**Conclusions and clinical implications:**

While PCNL with CBCT in a hybrid OR increases operative costs, it lowers SREs and unplanned care expenses. Even at a significantly lower hybrid OR utilization rate, total health care costs remain comparable, so PCNL-CBCT can facilitate predictable resource use and efficient care, with potential benefits for patients and health care systems.

**Patient summary:**

A new technique using CT (computed tomography) scans during surgery may help in more complete extraction of kidney stone fragments. Our study shows that even though this procedure is more expensive, it reduces the need for postoperative appointments, scans, emergency department visits and additional operations.

## Take Home Message

Intraoperative cone-beam computed tomography (CBCT) during percutaneous nephrolithotomy (PCNL) increases postoperative stone-free rates and reduces postoperative stone-related events (SREs), but widespread adoption depends on costs. Our results show that the higher procedural costs for CBCT-PCNL are offset by lower follow-up and SRE costs. Thus, CBCT-PCNL represents a clinically effective and financially viable strategy, and can also shift expenses from unplanned to planned care pathways.

## 1 Introduction

Achieving stone-free status is the primary goal of percutaneous nephrolithotomy (PCNL), as residual fragments (RFs) after surgery are associated with a higher likelihood of recurrent stone-related events (SREs) such as reinterventions, emergency department (ED) visits, and subsequent admissions [1], [2], [3], [4]. SREs not only impact patient outcomes but also contribute to higher health care costs [5], [6], [7]. Despite endoscopic and fluoroscopic confirmation of stone clearance during the procedure, stone-free status is falsely assumed in 20–50% of cases [3], [8], [9]. Intraoperative cone beam computed tomography (CBCT) may offer valuable advantages by enhancing detection of RFs and facilitating targeted removal during the same procedure [10].

A randomized controlled trial (RCT) demonstrated a statistically significant 15% improvement in 4-wk postoperative grade C (≤4 mm) stone-free rate on CT in favor of intraoperative CBCT versus PCNL without CBCT [9]. This finding suggests that CBCT is a promising technique for improving single-session PCNL outcomes and reducing future SREs and stone-related morbidity [11]. However, intraoperative CBCT use is associated with higher procedural costs because of the requirement for a hybrid operating room (OR), which may restrict broader adoption of CBCT in routine PCNL practice [12].

However, CBCT may offer potential health benefits and cost savings in the longer term. Higher stone-free rates could lead to lower costs because of fewer ED visits, hospital admissions, and repeat interventions. Therefore, it is crucial to evaluate the medium- and long-term costs associated with hybrid CBCT-PCNL in comparison to conventional PCNL. As no prior studies have assessed the total costs of hybrid versus conventional PCNL over extended follow-up, this analysis fills an important gap by including all procedural and hospital costs up to 18 mo. Our aim was to clarify whether the longer-term monetary benefits of hybrid PCNL outweigh its initial higher procedural costs.

## 2 Patients and methods

This study was conducted in accordance with the Declaration of Helsinki, with approval from the Medical Ethics Review Committee of University Medical Center Groningen (approval no. 2019/375), as reported elsewhere [9], [11], [13]. Written informed consent was obtained from all participants. Data collection and the study were monitored by an independent monitor. No external funding was received.

The data for the current study were retrospectively collected from a two-arm, parallel, nonblinded, single-center RCT conducted at a tertiary referral center specializing in complex urolithiasis in the Netherlands. A brief overview of the original RCT is provided below, followed by the methods used for the current cost analysis.

### 2.1 Original RCT procedures

Nonpregnant adults (≥18 yr) undergoing PCNL were eligible for inclusion in the original RCT. After confirming endoscopic and fluoroscopic stone-free status, patients were randomized intraoperatively (1:1, random blocks) to either intraoperative CBCT with additional extraction or procedure termination. The primary endpoint was grade C (≤4 mm) stone-free status on a 4-wk postoperative CT scan. Patients with bilateral stones undergoing staged PCNL were randomized per procedure. Detailed operative techniques and outcomes have been published previously [9], [11], [13].

### 2.2 Study design and cost analysis

The current analysis focused exclusively on hospital costs up to 18 mo postoperatively. At our center, regular follow-up includes CT and a consultation at 4 wk and 12 mo, with an additional 6-mo follow-up for high-risk cases. SREs included additional consultations, ED visits, admissions, drainage procedures, and reinterventions for ipsilateral RFs or stone recurrence, consistent with prior studies [14], [15].

Resource use per patient was linked to standardized costs from the Dutch Healthcare Institute (Supplementary Table 1) [16]. Costs included ED visits, hospital admissions, intensive care unit (ICU) stays, blood transfusions, diagnostics (eg, CT scans, ultrasound, X-rays), outpatient visits, telephone consultations, and additional interventions such as ureterorenoscopy (URS), PCNL, and double-J stent placement. Costs were categorized as initial OR, postoperative, follow-up, complication, or SRE-related costs.

Total costs were compared between the CBCT-PCNL and the regular PCNL group. The analysis was further stratified by stone-free status, dividing patients into three groups (stone-free, ≤4 mm RFs, and >4 mm RFs) to compare cost differences related to the residual stone burden.

If standardized cost data were unavailable (eg, for urology-specific procedures), costs were estimated from procedure duration multiplied by the reference OR price plus the cost of disposables, as in the model used by Patel et al. [12]. This model serves as the foundation for the reference prices determined by the Dutch Healthcare Institute. It accounts for various cost components, including the construction and size of the OR, staffing, and depreciation of equipment. Costs used based on the findings reported by Patel et al. [12] amount to €11.09/min for conventional ORs (92% utilization) and €23.34/min for hybrid ORs (43% utilization, as only procedures in which the fixed C-arm was used were included in the model calculation). Details of procedural times and disposable costs are provided in the Supplementary material.

For cases in the CBCT group who were stone-free at 4 wk, the costs of the 4-wk CT scan and consultation were omitted, as these are considered redundant for stone-free patients outside a research setting. PCNL reintervention costs were the same as those for the initial procedure in the control group. Costs for double-J stent and nephrostomy tube changes were not included. If a patient missed two consultations (no-show), whether in the outpatient clinic or via phone, a single consultation was recorded.

All costs were calculated in euros (€) with 2022 as the reference year for eventual adjustment using the annual consumer price index [17]. Given the relatively short time horizon, discounting was not applied. If patients were lost to follow-up and no additional records were found at the referring or regional hospital, it was assumed that no further costs were incurred.

### 2.3 Data collection and statistical analysis

Data were extracted from electronic patient records by a single observer (R.v.E.) and verified by a second observer (C.A.S.). All data were pseudonymized and securely stored in a Research Electronic Data Capture (REDCap) database in accordance with good clinical practice and the general data protection regulation. For patients lost to follow-up at our center or treated elsewhere, medical records were obtained from referring hospitals or regional centers.

Statistical assessment followed the intention-to-treat principle. Normality was assessed using the Kolmogorov-Smirnov test. Continuous variables are reported according to conventional statistical standards. Results for categorical variables are presented as the count and percentage. Group comparisons were performed using a χ^2^ test, Fisher’s exact test, independent t test, or Mann-Whitney U test, depending on the variable type and distribution. A *p* value of <0.05 was considered statistically significant. Analyses were conducted using R Studio v4.4.0 (R Foundation for Statistical Computing, Vienna, Austria).

Differences between endoscopic combined intrarenal surgery (ECIRS) and standard PCNL were evaluated for potential confounding by operative time. We conducted a sensitivity analysis to assess the impact of variation in OR utilization on costs, using the model developed by Patel et al. [12]. Nonparametric bootstrap analysis was performed to examine the uncertainty for the cost differences calculated.

## 3 Results

**Fig. 1:**
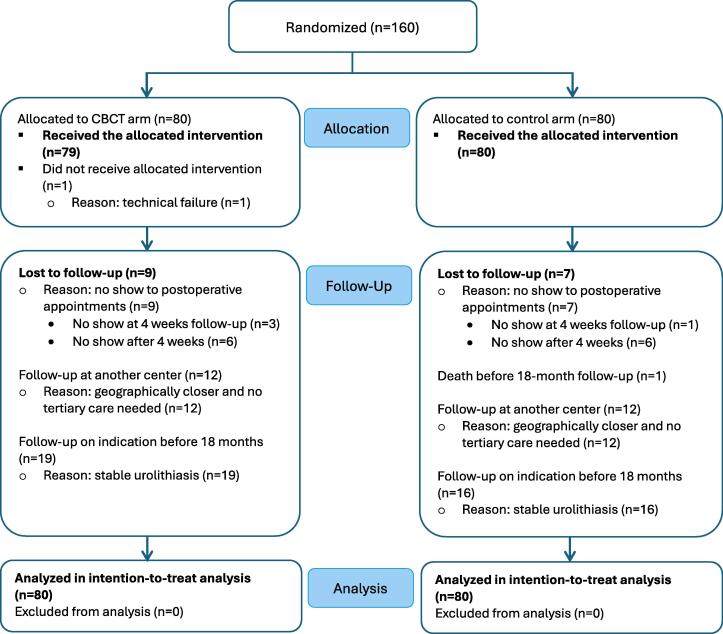
Flow diagram of the study inclusion process. CBCT = cone-beam computed tomography.

Between January 2020 and July 2023, 160 cases were randomized before early trial closure because interim analysis revealed that the predefined superiority margin was met. The final cohort included 149 patients, 11 of whom were randomized twice for bilateral procedures. One CBCT case lacked intraoperative imaging because of system failure. Fig. 1 shown an overview of the randomization and follow-up protocols.

There were no significant differences in demographics (Table 1) or postoperative complication rates (Table 2) between the groups. The median operating time was 13 min longer in the CBCT group than in the control group (77 min vs 64 min; Table 2). Operating time was not adjusted for ECIRS versus standard PCNL, as the proportion of ECIRS procedures was comparable between the groups (*p* = 0.34; Table 2).

**Table 1:** Demographics of the cohort (*n* = 149) by group

Variable	CBCT (*n* = 77)	Control (*n* = 72)
Median age, yr (IQR)	57 (46–67)	58 (47–67)
Sex, *n* (%)		
Male	37 (48.1)	47 (65.3)
Female	40 (51.9)	25 (34.7)
Median BMI, kg/m^2^ (IQR)	27 (25–32)	27 (23–31)
ASA score, *n* (%)		
1–2	48 (62.3)	47 (65.3)
3–4	29 (37.7)	25 (34.7)
Anatomic abnormality, *n* (%) a	27 (35.1)	17 (23.6)

ASA = American Society of Anesthesiologists physical status; BMI = body mass index; CBCT = cone-beam computed tomography; IQR = interquartile range.

a Abnormalities included paraplegia, spina bifida, horseshoe kidney, kidney transplant, ureteropelvic junction stenosis, urinary deviation, and others.

**Table 2:** Initial operation details and postoperative outcomes by study arm (160 procedures)

Parameter	Cone-beam CT (*n* = 80)	Control (*n* = 80)	*p* value a
Stone type, *n* (%)			
Single stone <20 mm	13 (16.3)	19 (23.8)	
Single stone >20 mm	6 (7.5)	6 (7.5)	
Multiple stones	49 (61.3)	43 (53.8)	
Partial staghorn stone	9 (11.3)	8 (10.0)	
Complete staghorn stone	3 (3.8)	4 (5.0)	
Surgery type, *n* (%)			
Percutaneous nephrolithotomy	50 (62.5)	44 (55.0)	
Endoscopic combined intrarenal surgery	30 (37.5)	36 (45.0)	
Median procedure duration, min (IQR)	77 (64–100)	64 (49–86)	
Cases with complications, *n* (%) b	19 (23.8)	23 (28.8)	
Healthcare resource utilization (*n*)			
Repeat percutaneous nephrolithotomy	7	9	0.60
Ureterorenoscopy	4	9	0.15
Double-J stent placement	4	9	0.49
Double-J stent removal	36	33	0.46
Procedures due to complication	2	2	1.00
Emergency department visits	19	24	0.29
Consultations	195	256	**0.016**
Hospital days	299	348	0.28
Intensive care unit days	4	23	0.40
CT scans	123	169	**<0.001**
Ultrasound	10	17	0.34
Abdominal X-ray	1	2	1.00
Red blood cells	4	2	0.32

CT = computed tomography.

a Mann-Whitney U test. Significant values are in bold font.

b All complications occurred within <30 d of surgery, except 1 complication in the control group, which was included in this analysis.

The cost per intervention was calculated as €4842 for PCNL-CBCT, €3118 for PCNL without CBCT, €835 for double-J stent placement, €2937 for URS, €3118 for PCNL reintervention, and €393 for double-J stent removal. The procedural costs and median procedure durations are presented in Supplementary Table 6.

**Fig. 2:**
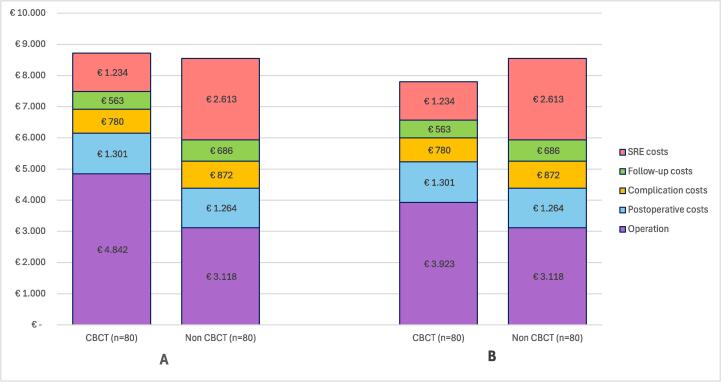
Breakdown of total mean costs per case by healthcare components. (A) Costs based on a utilization rate of 92% for the conventional operating room and 43% for the hybrid operating room. (B) Costs based on an equal utilization rate of 92% for both the conventional and hybrid operating rooms. CBCT = cone-beam computed tomography; SRE = stone-related event.

Analysis revealed lower resource utilization for CBCT group across nearly all categories in comparison to the control group (Table 2). The total number of consultations (195 vs 256; *p* = 0.016) and total number of CT scans (123 vs 169; *p* < 0.001) were significantly lower for the CBCT group. This led to lower mean follow-up costs in the CBCT group (€563 ± €299 vs €686 ± €229), although the difference did not reach conventional statistical significance (Fig. 2A).

The CBCT group had lower mean costs for complications (€780 ± €1823 vs €872 ± €2182) and SREs (€1234 ± €3327 vs €2613 ± €6338). SRE-related costs represented 14% of the total costs in the CBCT group, compared to 31% in the control group. Conversely, mean operative and postoperative costs were higher in the CBCT group (€6144 ± €509 vs €4382 ± €123), accounting for 70% of total costs versus 51% in the control group (Fig. 2A). Slightly higher postoperative costs in the CBCT group (€1264 ± 123 vs €1301 ± 509) were attributable to a single elective perioperative ICU admission requiring chronic ventilatory support. Overall, CBCT-PCNL was associated with 40.2% higher procedural costs but 38.3% lower follow-up, complication, and SRE-related costs. At 18 mo, the mean cumulative cost per case was €8720 ± €4120 in the CBCT group and €8553 ± €7323 in the control group, with a difference of €167 (1.9%) per case.

The utilization rate for the hybrid OR has a profound impact on the overall procedural cost. Deviations from the standard utilization rate of 43% can result in either cost savings or additional expenses, as illustrated in Supplementary Table 7. At a utilization rate of 47%, the total 18-mo follow-up costs for the CBCT group nearly match those for the control group (€8555 vs €8563). If the same utilization rate for both the ORs is used (92%), total costs for a CBCT-PCNL case is €752 less than the costs for a conventional PCNL case, as illustrated by Fig. 2B. In this scenario, total costs for the CBCT-PCNL group would be 8.8% lower than for the control group with standard PCNL.

In the CBCT group, total costs were €7988 ± €3232 for stone-free patients (*n* = 40), €8431 ± €3003 for cases with RFs ≤4 mm (*n* = 21), and €10 127 ± €4401 for cases with RFs >4 mm (*n* = 30). In the control group, the corresponding costs were €6509 ± €3738 (*n* = 33), €10 963 ± €8870 (*n* = 16), and €9640 ± €8961 (*n* = 30). Costs were approximately 5–40% lower for stone-free patients in comparison to those with RFs in both groups, although the difference was not statistically significant. Notably, a higher proportion of patients in the CBCT group achieved complete stone clearance.

**Fig. 3:**
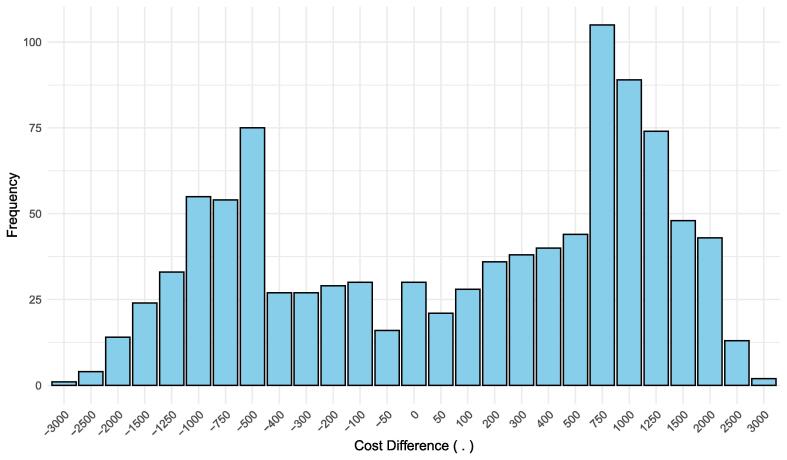
Bootstrap distribution histogram of resampled cost differences between the groups.

To assess the uncertainty of the cost difference between CBCT-PCNL and conventional PCNL, a bootstrap analysis with 1000 iterations was performed. This analysis yielded a mean cost difference of €128 ± €940 for utilization rates of 92% for the conventional OR and 43% for the hybrid OR. The bootstrap histogram (Fig. 3) illustrates the distribution of cost differences observed during the resampling process, with the highest frequencies observed between €750 and €1000. The 95% credibility interval for the cost difference ranged from −€1832 to €1736, which indicates that the difference is not statistically significant.

## 4 Discussion

This is, to the best of our knowledge, the first study to compare all hospital-related expenses for PCNL with intraoperative CBCT versus conventional PCNL over 18 mo. Our results show that despite higher initial procedural costs, intraoperative CBCT did not substantially increase total health care expenses. Using reference pricing, mean costs per case were €8720 for CBCT-PCNL and €8553 for conventional PCNL, with a difference of €167 (1.9%). Assuming an equal OR utilization rate of 92%, our calculations show that CBCT-PCNL is €752 (8.8%) less costly per procedure than conventional PCNL.

The economic impact of CBCT-PCNL largely depends on hybrid OR utilization [12]. At a 47% utilization rate, close to the 43% hospital average reported by Patel et al. [12], the total costs in both groups were comparable, which suggests potential cost neutrality. Although this threshold is based on single-center data, it indicates that higher OR utilization, which is increasingly achievable via multidisciplinary use by vascular, orthopedic, and trauma teams, may render CBCT-PCNL cost-neutral or even cost-saving. This finding supports broader adoption of PCNL with intraoperative CBCT, which could boost hybrid OR utilization and reduce costs [18], [19].

Moreover, CBCT-PCNL can shift health care spending towards planned elective care. Some 70% of costs were elective in the CBCT group versus 51% in the control group, while unplanned costs due to SREs were lower (14% vs 31%). Thus, higher upfront procedural costs may be offset by fewer unexpected interventions. Planned care improves workflows, reduces the staff burden, and enhances patient satisfaction, which are benefits that go beyond direct costs [20], [21]. These aspects are especially relevant in the current health care environment, in which both financial constraints and staffing shortages impact the delivery and quality of care [22]. The CBCT group also required fewer follow-up consultations and CT scans, which represents lower postoperative resource use.

RFs increased total costs in both groups, which emphasizes the value of complete stone clearance, although the differences were not statistically significant. The higher stone-free rate in the CBCT group probably contributed to fewer unplanned visits and SREs, which supports both clinical and economic benefits.

Our study has some limitations. Cost estimates relied on models that may overlook operational factors in conventional ORs, such as radiology staff and radiation protection measures, with possible underestimation of costs for the control group. While the 18-mo follow-up allows mid-term cost assessment, later SREs have not been captured. The single-center design limits generalizability, as protocols, surgical techniques, resources, and costs may vary across institutions. Results may also vary with surgeon experience.

Furthermore, the analysis was performed in the Dutch health care setting and used national reference prices that may not be representative of other health care systems. A study by Pietropaolo et al. [23] that was based on expert opinion revealed substantial international cost differences for endourological procedures, so direct translation of the absolute costs presented here to other health care contexts would be limited. Nonetheless, our findings suggest that better stone-free rates with CBCT-PCNL are likely to reduce resource utilization in the long term, and could potentially lead to cost savings irrespective of the specific set of prices applied.

The median operating time was 77 min for CBCT-PCNL and 64 min for conventional PCNL, with no statistically significant differences in stone complexity or procedure type (ECIRS vs PCNL) because of the RCT design [9]. However, operating times may be longer in routine CBCT-PCNL, as the most complex cases for which the likelihood of additional extraction seems highest are preferentially scheduled for CBCT-PCNL [24]. Consequently, our study may underestimate the procedural costs of CBCT-PCNL in regular practice.

Importantly, indirect societal costs, such as productivity loss and travel expenses, were not included. This omission is a major limitation, as urolithiasis often affects working-age patients. Previous studies have demonstrated that productivity loss is substantial, with approximately one-third of patients missing work for an average of 19 h per person [25]. The reduction in unplanned care and reinterventions in the CBCT-PCNL group may suggest that such losses could be less pronounced when improving stone-free rates with hybrid CBCT-PCNL. However, quantification of these costs would provide a more comprehensive estimate of the true economic burden of urolithiasis and its management.

No quality-of-life data were collected during this trial, which precludes cost-utility and incremental cost-effectiveness analyses. Ideally, future studies should have a multicenter design, involve larger sample sizes, include longer follow-up, collect quality-of-life data, and assess societal costs for a more comprehensive economic evaluation of hybrid PCNL with intraoperative CBCT.

Finally, our study was not powered for detection of cost differences, as the primary aim was to explore overall cost patterns and the feasibility of intraoperative CBCT, rather than to demonstrate cost superiority. The wide bootstrap confidence interval (−€1832 to €1736) reflects uncertainty in the cost estimates, so our finding of cost neutrality should be interpreted with caution. Nevertheless, the absence of a meaningful cost increase provides valuable insights and supports the economic viability of intraoperative CBCT during PCNL.

## 5 Conclusions

Our findings suggest that CBCT-PCNL increases procedural costs but reduces follow-up expenses, and thus results in no significant rise in total health care costs. At high hybrid-OR utilization, CBCT-PCNL may even be a cost-saving strategy. Despite limitations, such as the single-center design, the lack of power for detection of robust cost differences, and omission of non-hospital costs such as productivity loss, our study suggests that CBCT-PCNL lowers the health care burden and improves patient outcomes. Hybrid CBCT-PCNL therefore appears to be financially viable and clinically beneficial, and our results support wider adoption and future scientific scrutiny.

  ***Author contributions***: Chris A. Suijker had full access to all the data in the study and takes responsibility for the integrity of the data and the accuracy of the data analysis.

  *Study concept and design*: van Ee, Suijker, Kingma, van Asselt, Roemeling, van Oort.

*Acquisition of data*: van Ee, Suijker, Kingma.

*Analysis and interpretation of data*: van Ee, Suijker, van Asselt, Roemeling.

*Drafting of the manuscript*: van Ee, Suijker.

*Critical revision of the manuscript for important intellectual content*: van Ee, Suijker, Kingma, van Asselt, Roemeling, van Oort.

*Statistical analysis*: van Ee, Suijker.

*Obtaining funding*: None.

*Administrative, technical, or material support*: None.

*Supervision*: Kingma, van Asselt, Roemeling, van Oort.

*Other*: None.

  ***Financial disclosures:*** Chris A. Suijker certifies that all conflicts of interest, including specific financial interests and relationships and affiliations relevant to the subject matter or materials discussed in the manuscript (eg, employment/affiliation, grants or funding, consultancies, honoraria, stock ownership or options, expert testimony, royalties, or patents filed, received, or pending), are the following: None.

  ***Funding/Support and role of the sponsor*:** None.

  ***Acknowledgments***: We are grateful to Dolf Venhuizen for his contribution in determining the manufacturers’ charging costs for all the disposables used in this study.
