# Supplementary material for: Cost Comparison of Percutaneous Nephrolithotomy With and Without Intraoperative Cone-beam Computed Tomography: 18-month Postoperative Analysis
Source: Eur Urol Open Sci. 2025 Dec 18;83:125–32. doi: 10.1016/j.euros.2025.12.002 (PMC12775980; doi:10.1016/j.euros.2025.12.002)
Supplement: Supplementary Data 1 [file mmc1.docx]

**Supplementary material**

**Supplementary Table 1** Costing applied for analysis, price level 2022

|  | Costs |  | Source |
| --- | --- | --- | --- |
| Hybrid OR | € 23,34 | per minute | Guideline for Conducting Economic Evaluations in Healthcare (Version 2024) [1] |
| Conventional OR | € 11.09 | per minute |  |
| Hospital day | € 644,00 |  |  |
| ICU day | € 2727,00 |  |  |
| ED visit | € 258,00 |  |  |
| CT scan | € 188,00 |  |  |
| Ultrasound | € 118,00 |  |  |
| X-ray | € 82,17 |  |  |
| Outpatient visit | € 120,00 |  |  |
| Telephone consultation | € 120,00 |  | Regulation on Medical Specialist Care – NR/REC – 2306a – Article 24: Descriptions of Healthcare Activities [2] |
| Packed red blood cells | € 264,00 |  | Policy Rule on Availability Contribution Upon Request, Dutch Healthcare Authority [3] |

*OR operating room, ICU intensive care unit, ED emergency department, CT computed tomography*

**Calculation of procedural durations**

Because of heterogeneity in available data, several methods were used for the most reliable method of estimating median procedural duration. For the initial PCNL, room time was determined as the sum of the median operating time for the CBCT and control group, the median preparation time (induction and positioning), and the median post-procedure time. The median operating time for each group was determined by analyzing the first and last fluoroscopic images taken during the procedure. The median preparation time and post-procedure time was derived from data of 32 procedures of the CAPTURE study, as reported by Kingma et al [4]. Cost for the related disposables for PCNL are listed in Supplementary Table 2.

We have included a URS in all PCNL procedure costs, as it is routinely used in our practice. In ECIRS, the URS is employed for retrograde flexible ureterorenoscopy, whereas in PCNL, it is typically used in antegrade fashion through the nephrostomy tract to improve visualization of the collecting system for residual fragments or to retrieve fragments that have migrated and are difficult to reach with a nephroscope during intraoperative treatment.

In cases where a double J stent was placed as an exit strategy, the cost of a separate stent removal procedure was added, calculated as the sum of an outpatient clinic visit and the costs of the disposables required for double J stent removal (Supplementary Table 3).

The cost for a URS re-intervention was calculated with room times derived from anesthesiology record. Related disposables are listed in Supplementary Table 4. In cases where a double J stent was placed as an exit strategy, the cost of a separate stent removal procedure was added.

For double J stent placement, the same formula was used, with cost of required disposables listed in Supplementary Table 5.

The same pricing method was applied to other procedures performed due to complications, using available data such as procedure duration and required disposables to approximate the costs.

**Supplementary Table 2** Cost of the disposables including 9% VAT for a PCNL

|  | Costs |
| --- | --- |
| 2 x Sterile gown | € 8.35 |
| Drape PNL ioban | € 31.30 |
| 2 sterile covers | € 6.06 |
| Camera cover | € 3.17 |
| Neff set | € 133.60 |
| Trocar needle | € 28.78 |
| Lunderquist | € 42.16 |
| Endoguide | € 4.08 |
| Cystoscope | € 168.15 |
| Catheter | € 6.05 |
| Catheter bag 2 liters | € 2.66 |
| JJ catheter | € 101.85 |
| 2 x irrigation bag 3 liters NaCl 0.9% | € 9.29 |
| Disposable URS Pusen | € 721.05 |
| Instillagel | € 1.84 |
| 20cc syringe | € 0.16 |
| 10cc syringe | € 0.10 |
| Bladder syringe 60cc | € 0.41 |
| Single-use catheter ch 12 | € 0.59 |
| 3 bowl set | € 3.63 |
| Large bowl | € 1.28 |
| Drape | € 2.46 |
| 2 x sterile gauze | € 0.61 |
| Ultrasound gel | € 1.69 |
| Ureteral catheter | € 12.43 |
| ABP cap | € 23.20 |
| Scalpel 11 | € 0.96 |
| Tape strip | € 1.08 |
| Pot with white lid | € 0.31 |
| Trilogy probe | € 313.09 |
| Sterile/disposable needle holder | € 3.54 |
| Sterile/disposable scissors | € 2.59 |
| Three-way stopcock | € 0.47 |
| 2 x sterile gloves | € 2.79 |
| 10cc water for injections | € 0.20 |
| Suction connection tube | € 2.20 |
| Collection bags Trilogy | € 73.05 |
| Ultrasound cover | € 6.43 |
| Erbe water system | € 15.28 |
| Stone catcher set | € 80.88 |
| Vicryl rapide 4.0 | € 3.89 |
| 500cc NaCl 0.9% | € 1.00 |
| Contrast | € 8.80 |
|  |  |
| Total | **€ 1,831.56** |

**Supplementary Table 3** Cost of the disposables including 9% VAT for a Double J catheter removal.

|  | Costs |
| --- | --- |
| Cystoscope | € 168.15 |
| Packing pliers | € 102.92 |
| Instillagel | € 1.84 |
|  |  |
| Total | **€ 272.91** |

**Supplementary Table 4** Cost of the disposables including 9% VAT for a URS.

|  | Costs |
| --- | --- |
| TUR package | € 12.54 |
| Disposable URS Pusen | € 721.05 |
| Laser fiber 200µm | € 294.37 |
| Basket zero tip | € 251.29 |
| Sensor guidewire | € 41.40 |
| Ureteral catheter | € 12.43 |
| Contrast fluid | € 8.80 |
| Access sheath 13/11 35 cm | € 141.70 |
| Catheter | € 6.05 |
| Catheter bag 2 liters | € 2.66 |
| 2 sterile covers | € 6.06 |
| Instillagel | € 1.84 |
| 3 bowl set | € 3.63 |
| Erbe system | € 15.28 |
| 10cc syringe | € 0.10 |
| Water for injection | € 0.20 |
| Instillagel | € 1.84 |
| 500cc NaCl 0.9% | € 1.00 |
| Erbe system | € 15.28 |
| 2 x irrigation bag 3 liters NaCl 0.9% | € 9.29 |
| 2 x sterile gauze | € 0.61 |
| Sterile gown | € 4.17 |
| Camera cover | € 3.17 |
| JJ catheter | € 101.85 |
| Drape | € 2.46 |
| 2 x sterile gloves | € 2.79 |
| 10cc water for injections | € 0.20 |
|  |  |
| Total | **€ 1,662.04** |

**Supplementary Table 5** Cost of the disposables including 9% VAT for a Double J catheter placement.

|  | Costs |
| --- | --- |
| Ureteral catheter | € 12.43 |
| Sensor guidewire | € 41.40 |
| JJ catheter | € 101.85 |
| Cystoscopy pack | € 5.50 |
| Contrast | € 8.80 |
| Instillagel | € 1.84 |
| 3 bowl set | € 3.63 |
| 20cc syringe | € 0.16 |
| 1 x sterile gauze | € 0.31 |
| 500cc NaCl 0.9% | € 1.00 |
| Sterile gown | € 4.17 |
| Camera cover | € 3.17 |
| Drape | € 2.46 |
| 2 x sterile gloves | € 2.79 |
| Cystoscope | € 168.15 |
|  |  |
| Total | **€ 357.66** |

**Supplementary Table 6** Calculated cost applied for analyses

|  | **Median procedure duration** | **Cost per minute** | **Cost of disposables** | **Total Costs** |
| --- | --- | --- | --- | --- |
| PCNL with CBCT | 129 min | € 23.34 | € 1,831.56 | € 4,842.42 |
| PCNL | 116 min | € 11.09 | € 1,831.56 | € 3,118.00 |
| Double J placement | 43 min | € 11.09 | € 357.66 | € 834.53 |
| URS | 115 min | € 11.09 | € 1,662.04 | € 2,937.39 |
| Re-PCNL | 116 min | € 11.09 | € 1,831.56 | € 3,118.00 |
| Double J removal |  |  | € 272.91 | € 392.91 |

*CBCT cone beam computed tomography, URS ureterorenoscopy, PCNL percutaneous nephrolithotomy*

**Supplementary Table 7** Impact of hybrid OR utilization rate on costs

| Hybrid OR utilization rate (%) | Cost per minute (€) | Cost PCNL with CBCT (€) | Average total cost per CBCT case (€) | Average total cost per control case (€) | Cost difference per case (€) |
| --- | --- | --- | --- | --- | --- |
| 92 | 16.21 | 3,923 | 7,805 | 8,553 | - 752 |
| 80 | 17.17 | 4,046 | 7,929 | 8,553 | - 629 |
| 70 | 18.20 | 4,179 | 8,062 | 8,553 | - 496 |
| 60 | 19.60 | 4,360 | 8,243 | 8,553 | - 315 |
| 55 | 20.48 | 4,473 | 8,356 | 8,553 | - 202 |
| 47 | 22.02 | 4,672 | 8,555 | 8,553 | - 3 |
| 25 | 33.18 | 6,112 | 9,995 | 8,553 | 1,437 |
| 14 | 51.49 | 8,474 | 12,356 | 8,553 | 3,799 |

*CBCT cone beam computed tomography, OR operating room*

**References**

1. Zorginstituut Nederland. Richtlijn voor het uitvoeren van economische evaluaties in de gezondheidszorg (versie 2024). Diemen, The Netherlands: Zorginstituut Nederland; 2024. https://www.zorginstituutnederland.nl/publicaties/publicatie/2024/01/16/richtlijn-voor-het-uitvoeren-van-economische-evaluaties-in-de-gezondheidszorg

2. Nederlandse Zorgautoriteit. Regeling medisch-specialistische zorg - NR/REG-2306a. Artikel 2. Utrecht, The Netherlands: Nederlandse Zorgautoriteit; 2022. https://puc.overheid.nl/nza/doc/PUC_720738_22/1/#:~:text=Artikel%202

3. Nederlandse Zorgautoriteit. Beleidsregel Beschikbaarheidbijdrage op aanvraag, Utrecht, The Netherlands: Nederlandse Zorgautoriteit; 2022.

4. Kingma RA, De Jong IJ, Greuter MJW, Roemeling S. Cone beam computed tomography for detecting residual stones in percutaneous nephrolithotomy, a randomized controlled trial (CAPTURE) protocol. Trials 2021;22:805. https://doi.org/10.1186/s13063-021-05794-5
